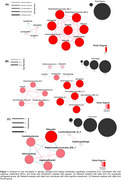# Oral Microbial Network Alterations in the Alzheimer’s Disease Continuum

**DOI:** 10.1002/alz70861_108925

**Published:** 2025-12-23

**Authors:** Isadora Crasnhak de Souza, Amanda Muliterno Domingues Lourenço de Lima, Marco De Bastiani, Wyllians D Borelli, Eduarda Letícia Klafke Ebert, Renan Antônio Barth, Joana Emilia Senger, Artur Francisco Schumacher‐Schuh, Emilio Hideyuki Moriguchi, Joao Senger, Neide Maria Bruscato, Berenice Maria Werle, Laisa Zanella, Lilian Vivian Netson, Ariele Detogni, Rafaela Ramalho Guerra, Andreza Francisco Martins, João Vitor Cardoso Barboza, Eduardo José Gaio, Eduardo R. Zimmer

**Affiliations:** ^1^ Universidade Federal do Rio Grande do Sul, Porto Alegre, Rio Grande do Sul Brazil; ^2^ Universidade Federal do Rio Grande do Sul, Porto Alegre, RS Brazil; ^3^ Universidade Federal de Ciências da Saúde de Porto Alegre, Porto Alegre, Rio Grande do Sul Brazil; ^4^ Hospital de Clínicas de Porto Alegre, Porto Alegre, RS Brazil; ^5^ Instituto Moriguchi, Veranópolis, Rio Grande do Sul Brazil; ^6^ Hospital de Clínicas de Porto Alegre, Porto Alegre, Rio Grande do Sul Brazil

## Abstract

**Background:**

Alzheimer’s disease (AD) is a progressive neurodegenerative disorder that ultimately leads to dementia. Emerging evidence suggests that oral dysbiosis may contribute to AD. The oral microbiota plays a crucial role in maintaining systemic health, and its imbalance has been associated with neurodegeneration. However, beyond the identification of individual taxa, the structure and dynamics of microbial communities—particularly their ecological interactions—remain poorly understood in AD. Here, we investigated abundance association networks of the oral microbiota across the AD continuum.

**Method:**

Saliva samples collected from 12 elderly individuals classified as cognitively unimpaired (CU), having mild cognitive impairment (MCI), or having Alzheimer’s disease (AD) were sequenced on the Illumina MiSeq™ platform, targeting the V3–V4 regions of the 16S rRNA gene. FASTQ files were processed using the DADA2 pipeline. Amplicon sequence variants (ASVs) were inferred, and taxonomic assignments were performed using the eHOMD 16S rRNA database. ASVs were normalized by rarefaction without replacement. Finally, normalized, centered log‐ratio transformed abundance data were used to construct genus‐level correlation networks for the CU, MCI, and AD groups.

**Result:**

In CU individuals, *Eikenella* maintained exclusively positive associations with other microbial taxa (Figure 1). However, these interactions were significantly reduced or shifted toward negative relationships in individuals with AD. Notably, one of the most pronounced changes was the weakened association between *Eikenella* and *Lachnospiraceae_G_3* in individuals with AD. Additionally, a positive relationship between *Eikenella* and *Peptostreptococcaceae_XIG_1* observed in CU shifted to a negative relationship in the AD group. These specific microbial associations were not observed in the MCI group.

**Conclusion:**

In summary, our results suggest a disruption in microbial synergy, which may reflect or potentially contribute to the underlying pathological mechanisms of AD. Network analysis may provide valuable insights into the dynamic changes within the oral microbiome across different stages of AD, thereby enhancing our understanding of the oral microbiome's role in neurodegenerative processes.